# Effectiveness of Rehabilitative Intervention on Pain, Postural Balance, and Quality of Life in Women with Multiple Vertebral Fragility Fractures: A Prospective Cohort Study

**DOI:** 10.3390/jfmk6010024

**Published:** 2021-03-03

**Authors:** Dalila Scaturro, Serena Rizzo, Valeria Sanfilippo, Valerio Giustino, Giuseppe Messina, Francesco Martines, Vincenzo Falco, Daniele Cuntrera, Antimo Moretti, Giovanni Iolascon, Giulia Letizia Mauro

**Affiliations:** 1 Department of Oncology and Stomatological Surgical Disciplines, University of Palermo, 90100 Palermo, Italy; dalila.scaturro@unipa.it (D.S.); serena.izzo@unipa.it (S.R.); valeria.sanfilippo@unipa.it (V.S.); giulia.letiziamauro@unipa.it (G.L.M.); 2 Program in Health Promotion and Cognitive Sciences, Department of Psychology, Educational Science and Human Movement, University of Palermo, 90100 Palermo, Italy; valerio.giustino@unipa.it; 3 Department of Psychology, Educational Science and Human Movement, University of Palermo, 90100 Palermo, Italy; giuseppe.messina@unipa.it; 4 PosturaLab Italia Research Institute, 90100 Palermo, Italy; 5 Istituto Euromediterraneo di Scienza e Tecnologia—IEMEST, 90100 Palermo, Italy; francesco.martines@unipa.it; 6 Bi.N.D. Department, Audiology Section, University of Palermo, 90100 Palermo, Italy; 7 Department of Economics, Statistics University of Palermo, 90100 Palermo, Italy; vincenzo.falco@unipa.it (V.F.); daniele.cuntrera@unipa.it (D.C.); 8 Department of Medical and Surgical Specialties and Dentistry, University of Campania “Luigi Vanvitelli”, 80138 Naples, Italy; giovanni.iolascon@unicampania.it

**Keywords:** pain, postural balance, quality of life, osteoporosis, vertebral fragility fractures, rehabilitation

## Abstract

Patients with vertebral fragility fractures often experience chronic pain, postural and balance disorders, and poor quality of life (QoL). Although several studies have investigated the role of rehabilitation in severe osteoporosis, the effectiveness of this intervention in patients with multiple vertebral fractures is poorly known. The aim of our longitudinal cohort study is to evaluate the effectiveness of rehabilitation, including postural training, resistance exercises, and visual stabilization exercises, for a 7-week period, on the pain, postural balance, and QoL of subjects with at least two vertebral fragility fractures receiving denosumab and vitamin D. We investigated, before (T0) and after (T1, at 7 weeks) rehabilitation, the following outcome measures on 28 patients: pain (Numerical Rating Scale (NRS)), self-perceived QoL (36-Item Short Form Survey (SF-36) and Mini-Osteoporosis Quality of Life Questionnaire (Mini-OQOL)), dizziness (Dizziness Handicap Inventory (DHI-I)), mobility (Timed-Up and Go (TUG) test), and instrumental posturographic assessment (FreeMed posturography system). At the end of the treatment, improvements of pain and QoL were recorded. Pain relief was highly obtained in patients with more than two vertebral fractures. Moreover, a significant functional improvement (TUG test) was found in those with two vertebral fractures, without any statistically significant change reported for other outcomes. Our findings suggest that combined intervention, including anti-osteoporotic drugs and postural rehabilitation, should be proposed to osteoporotic patients with multiple vertebral fractures.

## 1. Introduction

Osteoporosis is a systemic disease characterized by poor bone quality, reduced bone mass, and consequently, an increased risk of fragility fractures [1,2,3,4]. The prevalence of osteoporosis and fragility fractures is spreading worldwide with the increasing age of the global population. According to the International Osteoporosis Foundation (IOF) and the European Federation of Pharmaceutical Industry Association (EFPIA) [4], osteoporosis causes more than 8.9 million fractures annually worldwide—approximately 1000 per hour. The Italian Ministry of Health reported an annual incidence of 410,000 fragility fractures in Italy [2]. Vertebral fractures are the most common fragility fractures, occurring because of a low energy trauma, quantified as a force equivalent to a fall from a standing height or less, or in the absence of a recognized cause [2]. The main clinical complaints associated with these fractures include chronic pain, limited social participation, along with poor quality of life (QoL) [3], although less than one-third of patients with vertebral fragility fractures come to medical attention [5]. These fractures can impair mobility even when asymptomatic [6]. Indeed, postural alterations following vertebral fractures are associated with impaired gait pattern, contributing to poor physical performance [7,8]. It has been observed that the presence of these fractures modifies biomechanical forces on the spine, thus affecting intrinsic structural stability [9]. Vertebral fractures, particularly those affecting the thoracic spine, if not properly treated may lead to progressive increase in thoracic kyphosis which consequently displaces forward the centre of gravity, leading to an increased risk of both new vertebral fractures and falls [1,10]. If on one hand a relationship among mobility, fragility fractures, and falls [11] has been suggested, on the other, the role of vertigo and dizziness in terms of increased fall risk is poorly considered, although its prevalence ranges from 1.8% in young adults to more than 30% in elderly people [12]. Evaluating and treating vestibular and non-vestibular components of dizziness is recommended to avoid complications, such as falls [13]. However, data on the relationship between vertebral fragility fractures and postural control are controversial [14,15,16].

In the multidisciplinary approach to osteoporotic patients with fragility fractures, pharmacological therapy is a key intervention considering its efficacy in preventing incident fractures [17]. Antiresorptive drugs combined with calcium and vitamin D supplementation significantly reduce the risk of new vertebral fractures in subjects with a previous fracture, thus reducing the risk of increased hyperkyphosis and spinal misalignment because of re-fracture. In particular, denosumab seems to reduce pain and improve QoL in patients with vertebral fragility fractures [18,19,20] and has a remarkable compliance to the treatment [21]. Therefore, patients with fragility fractures treated with denosumab and vitamin D are an ideal cohort for analysing the effectiveness of a rehabilitation treatment on pain and functional issues, including postural alterations, and their influence on perceived quality of life in women with osteoporosis [22,23].

The aim of our study is to evaluate the effectiveness of rehabilitative intervention on pain, postural balance, and quality of life in postmenopausal women with at least two vertebral fragility fractures being treated with denosumab.

## 2. Materials and Methods

### 2.1. Study Design and Population

We carried out a prospective cohort study involving 52 women recruited at our Metabolic Bone Diseases Clinic of Physical Medicine and Rehabilitation. The study was led according to the principles of the Helsinki Declaration after the approval of the local ethics committee: Palermo Ethics Committee I (protocol number 05/2019 of the meeting of the 22 May 2019). The study protocol was fully explained, and written informed consent was obtained from each participant.

### 2.2. Participant Selection

Inclusion criteria were diagnosis of postmenopausal osteoporosis with at least two vertebral fragility fractures; ongoing pharmacological treatment with denosumab for at least 6 months combined with cholecalciferol supplementation; serum 25(OH)D3 ≥ 20 ng/mL. Non-ambulatory patients and those with ear diseases (medium and/or external ear cancers, infections, otosclerosis, active Meniere Disease, sudden hearing loss, and Enlarged Vestibular Aqueduct syndrome), cerebellopontine tumours, other neurological, and/or severe psychiatric diseases were excluded.

### 2.3. Intervention

All patients underwent a first evaluation before the treatment protocol and a second one after the end of the treatment period (7 weeks). All patients received the rehabilitation treatment led by a physiotherapist with expertise in postural rehabilitation, also integrating exercises to improve balance and walking pattern, resistance training, and visual stabilization exercises. This treatment protocol was administered 3 times per week, with 45 min sessions, for a total of 20 sessions, in a 7-week period.

### 2.4. Outcome Measures

#### 2.4.1. Numerical Rating Scale

After medical history collection and a standard musculoskeletal examination, pain, quality of life, posture, and balance at baseline were comprehensively assessed. Pain intensity was measured by the Numerical Rating Scale (NRS), a unidimensional measure of pain intensity in adults with chronic pain [24]; it is a segmented numeric version of the visual analogue scale (VAS) in which a respondent selects a whole number (0–10 integers) that best reflects the intensity of his/her pain. The common format is a horizontal bar or line where the 11-point numeric scale ranges from “0” representing “no pain” to “10” representing “pain as bad as you can imagine”.

#### 2.4.2. SF-36

Self-perceived QoL was investigated through the 36-Item Short Form Survey (SF-36) and Mini-Osteoporosis Quality of Life Questionnaire (Mini-OQOL), respectively. SF-36 consists of 36 items divided into eight health domains: general health (GH), physical functioning (PF), physical role (rP), body pain (BP), vitality (v), social functioning (SF), emotional role (re), and mental health (mH) [25]. Each domain was evaluated separately, and the total score ranged from 0 to 100 points, with a higher score indicating a better QoL.

#### 2.4.3. Mini-OQOL

The Mini-OQOL evaluates the QoL in patients affected by osteoporosis investigating five health areas (symptoms, emotional state, physical function, daily activities, and social activities) [26]. For each of the 10 questions, a mark between 1 and 7 is assigned. The total score of the questionnaire ranges from 10 to 70, while the marks of each area range from 2 to 14. For each item, a score of 1 corresponds to the worst possible function (extreme difficulty, permanent fear, and extreme anxiety), while a score of 7 is associated with the best function possible (absence of difficulty, fear, and anxiety).

#### 2.4.4. Timed-Up and Go 

Postural and balance were clinically assessed by the Timed-Up and Go (TUG)-Test [27], while the instrumental assessment of the outcomes was performed by the FreeMed posturography system. The TUG-Test was used to assess balance and fall risk. Participants were instructed to get up from a chair, walk up to a sign marked on the floor 3 m ahead on the chair, turn around in circles, walk up to the chair, and sit down. The time of performance was measured in seconds, and lower values indicate better balance control and lower fall risk.

#### 2.4.5. Dizziness Handicap Inventory (DHI)

Dizziness burden was investigated by the Dizziness Handicap Inventory in Italian version (DHI-I) [28,29]. DHI-I is a questionnaire formed by 25 items that measure the impact of dizziness on functional (9 items), emotional (8 items), and physical domains (7 items). Each answer is assigned: 4 points for “yes”, 2 points for “often”, and 0 points for “no”; the score of the questionnaire ranges from 0 to 100 and is used as the clinical base for the evaluation of clinical dizziness severity.

#### 2.4.6. Instrumental Assessment of Balance and Gait

Posturographic assessment was registered in a sound-isolated booth using the FreeMed posturography system, including the FreeMed baropodometric platform and the FreeStep v.1.0.3 software. The system was set to sample postural sway at 100 Hz. The sensors, coated with 24 K gold, guarantee repeatability and reliability of the instrument (produced by Sensor Medica, Guidonia Montecelio, Roma, Italy). The posturographic analysis was led by a stabilometric platform. Baropodometric examination was performed asking patients to maintain their static position without shoes, with their arms by their sides, head, and consequently, sight in neutral position with open eyes for 5 s; they also underwent a static analysis (stabilometric examination) maintaining standing position with feet placed together with an angle of 30 grades between right and left heels at 2 cm apart, firstly, with open eyes for 51.2 s, and after that, with closed eyes for 51.2 s too, in order to analyse the time and frequency of oscillations and self-adjustments of the patient excluding the visual input. In baropodometry, the following parameters were considered: pressure of right and left foot on the ground (kg and %), pressure load of both left and right forefoot and backfoot (%), and the relation between backfoot and forefoot (%). Additionally, the maximum pressure point and the medium pressure point (gr/cm^2^) between feet and for each foot, the plantar surface of both feet and the superficial extension of the backfoot and forefoot of each foot (cm^2^), the podalic angle, and axe were measured. In stabilometric examination, we considered the bundle length (mm) that is the size of the trait designed by the oscillation of the centre of pressure (CoP) during the test; the ellipse surface (mm^2^) is the area including 90% of the trait of the CoP; X-medium (mm) indicates the medium position maintained in frontal plane during the lateral oscillations, and Y-medium (mm) is the medium point of the centre of gravity on the sagittal plane during anterior-posterior oscillations; ΔX (mm) is the range of right-left oscillations, and ΔY (mm) is the range of anterior–posterior oscillations. All patients also underwent a dynamic biomechanical evaluation of gait and were asked to walk along the full length of the platform10 times, with their sight in neutral position. We analysed the maximum load and medium load (Kg and %), plantar maximum and medium surface (Kg and %), the maximum pressure point of feet and medium pressure point of feet (gr/cm^2^), the step length and the half-step length (mm), the medium walking velocity (mm/s), the number of steps per minute, the time permanence of each foot on the ground (ms), the total duration time of the test (s), and the number of footprints scanned. Moreover, for each footprint in every phase of the step, data related to load (%), surface (mm^2^) of the feet toes, metatarsal bones, and medial and lateral plantar arch were analysed.

### 2.5. Statistical Analysis

All outcome measures were assessed after the last session of treatment (T1, 7-week follow-up). All data were presented as the mean ± standard deviation (SD) for continuous variables and as the median (interquartile range) for ordinal variables. All analyses are performed using R software (R Core Team, Vienna, Austria, 2013). Differences in the demographic characteristics; parameters of stabilometric, static, and dynamic baropodometry; and the questionnaires that were administered between the first and the second examination (T0 and T1) were compared using a Student’s *t* test for continuous variables and Mood’s test for ordinal variables. Values of *p* < 0.05 were considered statistically significant.

## 3. Results

**Figure 1:**
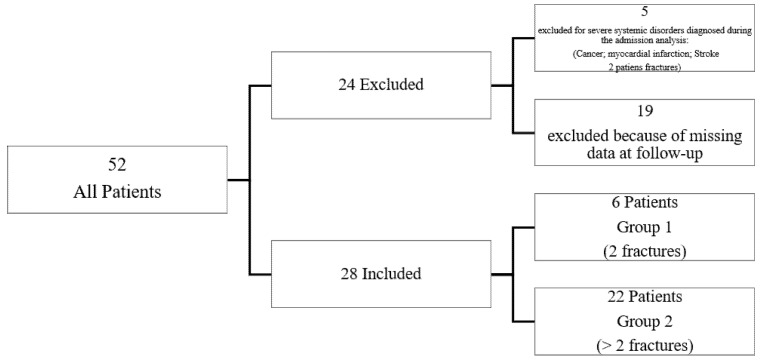
Selection of the study population.

Out of 52 women included, five of them were excluded because of severe systemic disorders diagnosed during the study (one patient had cancer, one patient had myocardial infarction and another one had a stroke, and two patients had a fracture). Then, 19 of them were excluded because of missing data at follow-up (T1). Finally, 28 women completed the rehabilitation protocol (six patients with two vertebral fractures, Group 1, and 22 patients with more than two vertebral fractures, Group 2) (Figure 1).

The baseline characteristics of study population are reported in Table 1. The age range was 55–77 years with a mean age of 66.5 ± 5.3 years. At T1, statistically significant improvements of NRS and SF-36 were reported in our cohort. For the within-group analysis, significant improvements for NRS and TUG were reported in Group 2 and Group 1, respectively. For the between-group analysis, a significant difference was reported at baseline TUG (Table 1).

In static baropodometry, there were no statistically significant differences between T0 and T1 in all patients and both Group 1 and Group 2 for any of the parameters assessed, except for the Right Pod Degree in Group 1 (Table 2).

Additionally, in both the stabilometric analysis and dynamic evaluation, no statistically significant differences between time points for all patients and in both Group 1 and Group 2 for any of the parameters assessed were found. All patients completed the treatment protocol, and no adverse event was reported (Table 3 and Table 4).

## 4. Discussion

To the best of our knowledge, this is the first study to investigate the effectiveness of a combined approach, including denosumab and vitamin D administration, and rehabilitation interventions in patients with multiple vertebral fractures in terms of key outcomes, including pain, mobility, postural control, and quality of life. Our findings suggest that this treatment strategy might be effective for pain relief, particularly in osteoporotic women with more than two vertebral fractures. Moreover, the same intervention might also improve mobility in women with two vertebral fragility fractures. It should be underlined that the rehabilitation treatment was well tolerated, despite the severity of bone fragility in our population.

Osteoporotic fractures have significant detrimental effects in terms of pain, mobility limitations and postural balance, and QoL [30,31]. Recently, Stanghelle et al. [32] claimed that poor QoL was significantly associated with both limitations of physical functioning and higher pain intensity in women with vertebral fragility fractures, supporting the main role of pain management and therapeutic exercise for this population.

Pain control could promote adherence to rehabilitation treatment with putative improvement of muscle impairments that are commonly observed in patients with severe osteoporosis, including those with multiple vertebral fractures [33,34]. In a real practice prospective study, including osteoporotic women with vertebral fractures (50% of participants had at least three fractures) and chronic back pain, 1-year denosumab administration resulted to be effective in reducing back pain related disability and QoL after 6 months of treatment [20]. The analgesic effects of this drug were hypothesized through negative modulation of the nuclear factor-κB (NF-κB), via inhibition of the RANK/RANKL pathway, with consequent inhibition of osteoclasts activity that reduces local acidification, thus reducing bone pain [35]. Denosumab was also demonstrated to reduce bone marrow edema that is usually painful [36].

More recently, a putative role of the OPG/RANK/RANKL pathway has been suggested in the pathogenesis of skeletal muscle wasting. An experimental study demonstrated that the systemic injection of OPG restores muscle strength and improves muscle quality in mice models of muscular dystrophy [37]. Furthermore, muscle RANK regulates calcium ion storage and sarco/endoplasmic reticulum Ca2+ATPase (SERCA) activity of fast-twitch muscle fibres, which are involved in fall prevention [38,39]. From a clinical perspective, a significant lower incidence of falls compared to placebo (−21%) has been reported in randomized controlled trials investigating the efficacy of denosumab for the prevention of fragility fractures [40].

Concerning mobility impairment, our results are in line with those reported by a recent Cochrane Systematic Review, which found moderate-quality evidence supporting the efficacy of exercise in improving physical performance, specifically TUG, in patients with vertebral fractures [41]. Moreover, a recent study suggests that thoracic hyperkyphosis is significantly associated with increased TUG time in older women with vertebral fractures [42]. In our study, postural parameters do not seem to be affected by rehabilitation intervention, and this is probably due to the short period of treatment. Our findings are in line with those of some papers reporting no significant correlations between the instrumental parameters of postural balance and vertebral fractures in osteoporotic patients [14,43]. On the contrary, supervised high-intensity, progressive resistance, and impact training (HiRIT) carried out for 8 months, twice-weekly, including deadlift, squat, overhead press, and jumping chin-ups with drop landings, improved thoracic kyphosis compared to low-intensity exercise training without increasing the risk of new vertebral fractures or worsening existing vertebral deformities in postmenopausal women affected by osteoporosis [44]. However, in the population enrolled, only a few patients had an history of vertebral fractures (about one-third). Our data support the safety of therapeutic exercise even in a population with severe bone fragility, suggesting that concerns are overly conservative.

However, our study has some limitations, including the small sample size, the lack of core muscle strength evaluation, and the lack of a control group.

It can be speculated that the synergy between pharmacotherapy and rehabilitation treatment might be effective in reducing pain, thus improving the QoL of women affected by severe osteoporosis. In this context, therapeutic exercise, including postural techniques and resistance training, might be effective in improving postural balance by increasing the strength of the trunk muscles, thus reducing the risk of hyperkyphosis, falls, and incident fractures [45].

These findings can inform clinicians and stakeholders about the importance of combined approaches in women with multiple vertebral fragility fractures, also considering the association among better QoL, pain relief, and functional improvement in this population [46].

## 5. Conclusions

In this study, performed on a cohort of women suffering from osteoporosis with multiple vertebral fragility fractures, it seems that a combined approach, including antiresorptive drugs and rehabilitation intervention, might reduce pain relief and improve QoL, although further studies comparing this approach to pharmacological treatment alone, or therapeutic exercise alone, might improve the quality of evidence on this topic. Our findings suggest that this intervention is useful and should also be proposed to osteoporotic patients with severe conditions, including those with multiple vertebral fractures.

## Figures and Tables

**Table 1:** Baseline characteristics and values of clinical outcome measures before and after treatment.

	All Patients (*n* = 28)			Group 1: 2 Fractures (*n* = 6)			Group 2: >2 Fractures (*n* = 22)			*p*-Value between-Group in T0
	T0	T1	*p*-Value	T0	T1	*p*-Value	T0	T1	*p*-Value	
Age (years)	66.5 ± 5.3			65.5 ± 7.6			66.8 ± 4.6			0.71
BMI (kg/m^2^)	26.5 ± 4.0			24.0 ± 4.6			27.2 ± 3.6			0.16
NRS	6.5 (1.0)	5.0 (2.0)	<0.05	5.0 (2.7)	3.0 (0.7)	0.54	7.0 (1.7)	6.0 (1.7)	<0.05	0.65
SF-36	42.2 ± 15.8	50.4 ± 13.2	<0.05	45.0 ± 18.5	58.0 ± 10.4	0.17	41.5 ± 15.4	48.3 ± 1.2	0.12	0.48
Mini-OQOL	47.0 ± 10.0	51.9 ± 9.4	0.06	52.7 ± 8.3	55.5 ± 9.5	0.59	45.5 ± 10.0	51.0 ± 9.3	0.07	0.11
DHI-I	38.3 ± 25.5	32.7 ± 22.1	0.38	31.5 ± 25.5	29.2 ± 25.2	0.88	40.2 ± 25.8	33.7 ± 21.8	0.37	0.68
TUG	13.0 (2.0)	11.5 (2.5)	0.78	12.0 (0.0)	10.5 (1.0)	<0.05	14.0 (1.7)	12.7 (2.5)	0.76	0.02

**Abbreviations.** BMI: Body Mass Index; NRS: numeric rating scale; SF-36: 36-Item Short Form Survey; Mini-OQOL: Mini-Osteoporosis Quality of Life Questionnaire; DHI-I: Dizziness Handicap Inventory—Italian version; TUG: timed up and go.

**Table 2:** Posturographic analysis.

	All Patients (*n* = 28)			Group 1: 2 Fractures (*n* = 6)			Group 2: >2 Fractures (*n* = 22)		
	T0	T1	*p*-Value	T0	T1	*p*-Value	T0	T1	*p*-Value
Left surface cm^2^	104.6 ± 21.5	101.0 ± 25.2	0.57	97.7 ± 21.0	102.8 ± 16.0	0.64	106.5 ± 21.7	100.5 ± 27.5	0.43
Right surface cm^2^	104.6 ± 20.1	102.6 ± 19.5	0.71	103.8 ± 22.1	101.7 ± 11.5	0.84	104.8 ± 20.1	102.9 ± 21.4	0.76
Left Forefoot Surf. cm^2^	56.8 ± 13.6	54.4 ± 17.1	0.56	54.5 ± 11.9	58.7 ± 11.9	0.56	57.4 ± 14.3	53.2 ± 18.3	0.40
Right Forefoot Surf. cm^2^	56.9 ± 12.8	55.1 ± 13.4	0.61	58.7 ± 13.3	57.7 ± 10.4	0.89	56.5 ± 12.9	54.5 ± 14.2	0.63
Left Backfoot Surf. cm^2^	47.7 ± 9.3	46.6 ± 9.5	0.66	43.0 ± 9.2	44.0 ± 7.0	0.84	49.0 ± 9.1	47.3 ± 10.1	0.56
Right Backfoot Surf. cm^2^	47.8 ± 9.7	47.4 ± 8.1	0.87	45.5 ± 9.8	44.0 ± 3.2	0.73	48.5 ± 9.9	48.4 ± 8.8	0.97
Left Load %	49.9 ± 5.7	48.5 ± 6.0	0.39	47.8 ± 7.2	50.5 ± 8.7	0.58	50.4 ± 5.3	48.0 ± 5.1	0.13
Right Load %	50.1 ± 5.7	51.5 ± 6.0	0.39	52.2 ± 7.2	49.5 ± 8.7	0.58	49.6 ± 5.3	52.0 ± 5.1	0.13
Left Forefoot Load %	42.6 ± 8.0	42.3 ± 11.9	0.92	45.5 ± 3.2	51.0 ± 12.8	0.35	41.8 ± 8.8	39.9 ± 10.8	0.53
Right Forefoot Load %	44.9 ± 10.4	44.9 ± 10.7	0.99	45.7 ± 5.0	49.5 ± 10.1	0.43	44.7 ± 11.6	43.6 ± 10.8	0.75
Left Backfoot Load %	57.4 ± 8.0	57.7 ± 11.9	0.92	54.5 ± 3.2	49.0 ± 12.8	0.35	58.2 ± 8.8	60.1 ± 10.8	0.53
Right Backfoot Load %	55.1 ± 10.4	55.1 ± 10.7	0.99	54.3 ± 5.0	50.5 ± 10.1	0.43	55.3 ± 11.6	56.4 ± 10.8	0.75
CoP X Coord	13.9 ± 2.4	14.2 ± 2.2	0.67	12.6 ± 1.8	13.0 ± 2.7	0.78	14.3 ± 2.4	14.5 ± 1.9	0.74
CoP Y Coord	15.9 ± 2.2	16.2 ± 2.2	0.59	14.3 ± 0.7	14.7 ± 2.5	0.74	16.3 ± 2.3	16.6 ± 1.9	0.64
Left Pod Degree	6.3 ± 4.0	7.5 ± 3.7	0.28	4.8 ± 3.2	6.8 ± 2.6	0.26	6.7 ± 4.2	7.6 ± 4.0	0.47
Right Pod Degree	7.6 ± 4.8	9.4 ± 5.5	0.19	4.8 ± 4.1	10.0 ± 3.7	0.04	8.4 ± 4.8	9.3 ± 5.9	0.58

**Table 3:** Stabilometric analysis.

	**Open Eyes**								
	**All Patients (*n* = 28)**			**Group 1: 2 Fractures (*n* = 6)**			**Group 2: >2 Fractures (*n* = 22)**		
	**T0**	**T1**	***p*-Value**	**T0**	**T1**	***p*-Value**	**T0**	**T1**	***p*-Value**
Surface ellipse cm	129.9 ± 101.1	152.9 ± 205.8	0.60	87.8 ± 61.9	89.8 ± 56.3	0.95	141.4 ± 107.6	170.1 ± 228.6	0.60
Bundle length mm	493.3 ± 154.3	529.0 ± 170.4	0.42	475.5 ± 175.9	582.2 ± 143.6	0.28	498.2 ± 152.1	514.5 ± 177.2	0.75
Oscillation maximum	2.0 ± 1.3	2.0 ± 0.6	0.79	1.6 ± 0.3	1.9 ± 0.4	0.19	2.2 ± 1.5	2.0 ± 0.7	0.61
Velocity average mm/s	10.1 ± 3.1	10.7 ± 3.4	0.48	9.7 ± 3.6	11.8 ± 2.8	0.30	10.2 ± 3.1	10.4 ± 3.5	0.82
X average	0.1 ± 7.4	−1.5 ± 9.6	0.50	−1.8 ± 7.9	3.0 ± 11.0	0.41	0.6 ± 7.4	−2.7 ± 9.0	0.19
Y average	−17.3 ± 12.9	−17.0 ± 11.7	0.93	−164 ± 11.3	−11.9 ± 13.9	0.55	−17.6 ± 13.6	−18.4 ± 11.0	0.82
Standard Deviation X	2.2 ± 1.0	2.5 ± 1.3	0.30	1.6 ± 0.8	2.1 ± 0.5	0.21	2.4 ± 1.0	2.7 ± 1.4	0.47
Standard Deviation Y	2.8 ± 1.2	2.7 ± 1.5	0.95	2.6 ± 0.9	2.2 ± 1.2	0.54	2.8 ± 1.3	2.9 ± 1.6	0.86
	**Closed Eyes**								
	**All Patients (*n* = 28)**			**Group 1: 2 Fractures (*n* = 6)**			**Group 2: >2 Fractures (*n* = 22)**		
	**T0**	**T1**	***p*-Value**	**T0**	**T1**	***p*-Value**	**T0**	**T1**	***p*-Value**
Surface ellipse cm	248.4 ± 370.9	192.3 ± 205.1	0.49	148.3 ± 134.3	102.4 ± 101.4	0.52	275.8 ± 411.0	216.9 ± 220.7	0.56
Bundle length mm	536.5 ± 142.6	560.8 ± 209.9	0.61	559.0 ± 172.0	516.9 ± 82.3	0.61	530.4 ± 137.6	572.8 ± 233.1	0.47
Oscillation maximum	5.6 ± 4.3	6.3 ± 5.1	0.53	5.0 ± 2.9	8.2 ± 8.4	0.41	5.7 ± 4.6	5.9 ± 3.9	0.91
Velocity average mm/s	10.8 ± 2.9	11.3 ± 4.1	0.61	11.3 ± 3.5	10.4 ± 1.5	0.60	10.6 ± 2.8	11.5 ± 4.6	0.45
X average	0.3 ± 8.4	−1.5 ± 8.7	0.42	−3.5 ± 12.1	2.2 ± 8.0	0.36	1.4 ± 7.1	−2.6 ± 8.8	0.11
Y average	−17.2 ± 12.7	−16.5 ± 10.3	0.83	−13.5 ± 9.0	−13.3 ± 10.9	0.97	−18.2 ± 13.5	−17.4 ± 10.2	0.83
Standard Deviation X	3.0 ± 2.8	2.5 ± 1.6	0.37	2.1 ± 1.1	1.9 ± 1.3	0.79	3.2 ± 3.0	2.6 ± 1.6	0.39
Standard Deviation Y	3.0 ± 2.0	3.2 ± 2.2	0.74	3.0 ± 1.9	2.3 ± 1.8	0.51	3.1 ± 2.0	3.5 ± 2.3	0.51

**Table 4:** Dynamic evaluation.

	All Patients (*n* = 28)			Group 1: 2 Fractures (*n* = 6)			Group 2: >2 Fractures (*n* = 22)		
	T0	T1	*p*-Value	T0	T1	*p*-Value	T0	T1	*p*-Value
Length Left Gait Line mm	187.6 ± 36.5	186.9 ± 23.9	0.93	162.7 ± 63.5	175.7 ± 26.6	0.66	194.5 ± 23.0	190.0 ± 22.8	0.52
Length Right Gait Line mm	184.3 ± 44.8	186.5 ± 18.1	0.80	177.5 ± 40.7	184.7 ± 9.9	0.69	186.1 ± 46.6	187.0 ± 19.9	0.93
Left Forefoot Load %	60.4 ± 6.2	62.9 ± 5.4	0.11	60.0 ± 6.7	65.2 ± 5.1	0.17	60.5 ± 6.2	62.3 ± 5.5	0.31
Right Forefoot Load %	61.7 ± 5.8	61.0 ± 5.8	0.63	65.0 ± 5.7	60.3 ± 5.6	0.19	60.8 ± 5.6	61.1 ± 6.0	0.86
Left Backfoot Load %	39.6 ± 6.2	37.1 ± 5.4	0.11	40.0 ± 6.7	34.8 ± 5.1	0.17	39.5 ± 6.2	37.7 ± 5.5	0.31
Right Backfoot Load %	38.3 ± 5.8	39.0 ± 5.8	0.63	35.0 ± 5.7	39.7 ± 5.6	0.19	39.2 ± 5.6	38.9 ± 6.0	0.86
Left side Load %	50.7 ± 6.6	50.4 ± 6.3	0.85	53.5 ± 7.7	53.3 ± 7.8	0.97	49.9 ± 6.2	49.5 ± 5.7	0.84
Right side Load %	48.7 ± 5.6	50.0 ± 5.5	0.39	47.7 ± 5.8	53.8 ± 5.5	0.09	49.0 ± 5.7	48.9 ± 5.1	0.98

## Data Availability

The data presented in this study are available on request from the corresponding author. The data are not publicly available due to privacy.

## References

[1] Cultrera P., Pratelli E., Petrai V., Postiglione M., Zambelan G., Pasquetti P. (2010). Evaluation with stabilometric platform of balance disorders in osteoporosis patients. A proposal for a diagnostic protocol. Clin. Cases Miner. Bone Metab..

[2] Tarantino U., Iolascon G., Cianferotti L., Masi L., Marcucci G., Giusti F., Marini F., Parri S., Feola M., Rao C. (2017). Clinical guidelines for the prevention and treatment of osteoporosis: Summary statements and recommendations from the Italian Society for Orthopaedics and Traumatology. J. Orthop. Traumatol..

[3] Ferreira M.L., March L. (2019). Vertebral fragility fractures—How to treat them?. Best Pract. Res. Clin. Rheumatol..

[4] Hernlund E., Svedbom A., Ivergård M., Compston J., Cooper C., Stenmark J., McCloskey E.V., Jönsson B., Kanis J.A. (2013). Osteoporosis in the European Union: Medical management, epidemiology and economic burden. A report prepared in collaboration with the International Osteoporosis Foundation (IOF) and the European Federation of Pharmaceutical Industry Associations (EFPIA). Arch. Osteoporos..

[5] Clark E.M., Gooberman-Hill R., Peters T.J. (2016). Using self-reports of pain and other variables to distinguish between older women with back pain due to vertebral fractures and those with back pain due to degenerative changes. Osteoporos. Int..

[6] Imai T., Tanaka S., Kawakami K., Miyazaki T., Hagino H., Shiraki M., A-TOP (Adequate Treatment of Osteoporosis) Research Group (2017). Health state utility values and patient-reported outcomes before and after vertebral and non-vertebral fractures in an osteoporosis clinical trial. Osteoporos. Int..

[7] Balzini L., Vannucchi L., Benvenuti F., Benucci M., Monni M., Cappozzo A., Stanhope S.J. (2003). Clinical characteristics of flexed posture in elderly women. J. Am. Geriatr. Soc..

[8] Lorbergs A.L., Murabito J.M., Jarraya M., Guermazi A., Allaire B.T., Yang L., Kiel D.P., Cupples L.A., Bouxsein M.L., Travison T.G. (2017). Thoracic Kyphosis and Physical Function: The Framingham Study. J. Am. Geriatr. Soc..

[9] Izzo R., Guarnieri G., Guglielmi G., Muto M. (2013). Biomechanics of the spine. Part I: Spinal stability. Eur. J. Radiol..

[10] Iolascon G., Moretti A., Giamattei M.T., Migliaccio S., Gimigliano F. (2015). Prevalent fragility fractures as risk factor for skeletal muscle function deficit and dysmobility syndrome in post-menopausal women. Aging Clin. Exp. Res..

[11] Iolascon G., de Sire A., Calafiore D., Benedetti M.G., Cisari C., Letizia Mauro G., Migliaccio S., Nuti R., Resmini G., Gonnelli S. (2020). Multifactorial Assessment of Risk of Falling in 753 Post-Menopausal Women: A Multicenter Cross-Sectional Study by the Italian Group for the Study of Metabolic Bone Diseases. Clin. Interv. Aging.

[12] Fernández L., Breinbauer H.A., Delano P.H. (2015). Vertigo and Dizziness in the Elderly. Front. Neurol..

[13] Jahn K. (2019). The Aging Vestibular System: Dizziness and Imbalance in the Elderly. Adv. Otorhinolaryngol..

[14] Falossi F., Notarstefano C., Miccoli M., Pelagatti A., Raffaetà G. (2020). Balance impairment and fragility vertebral fractures. Clin. Ter..

[15] Ishikawa Y., Miyakoshi N., Kasukawa Y., Hongo M., Shimada Y. (2009). Spinal curvature and postural balance in patients with osteoporosis. Osteoporos. Int..

[16] Greig A.M., Bennell K.L., Briggs A.M., Wark J.D., Hodges P.W. (2006). Balance impairment is related to vertebral fracture rather than thoracic kyphosis in individuals with osteoporosis. Osteoporos. Int..

[17] Iolascon G., Moretti A., Toro G., Gimigliano F., Liguori S., Paoletta M. (2020). Pharmacological Therapy of Osteoporosis: What’s New?. Clin. Interv. Aging.

[18] Jin Y.Z., Lee J.H., Xu B., Cho M. (2019). Effect of medications on prevention of secondary osteoporotic vertebral compression fracture, non-vertebral fracture, and discontinuation due to adverse events: A meta-analysis of randomized controlled trials. BMC Musculoskelet. Disord..

[19] Moretti A., Gimigliano F., Di Pietro G., Gimigliano R., Iolascon G. (2015). Back pain-related disability and quality of life in patients affected by vertebral fractures: Data from baseline characteristics of population enrolled in Denosumab In Real Practice (DIRP). Aging Clin. Exp. Res..

[20] Moretti A., de Sire A., Curci C., Toro G., Gimigliano F., Iolascon G. (2018). Effectiveness of denosumab on back pain-related disability and quality-of-life in patients with vertebral fragility fractures. Curr. Med. Res. Opin..

[21] Migliaccio S., Francomano D., Romagnoli E., Marocco C., Fornari R., Resmini G., Buffa A., Di Pietro G., Corvaglia S., Gimigliano F. (2017). Persistence with denosumab therapy in women affected by osteoporosis with fragility fractures: A multicenter observational real practice study in Italy. J. Endocrinol. Investig..

[22] Brox W.T., Roberts K.C., Taksali S., Wright D.G., Wixted J.J., Tubb C.C., Patt J.C., Templeton K.J., Dickman E., Adler R.A. (2015). The American Academy of Orthopaedic Surgeons Evidence-Based Guideline on Management of Hip Fractures in the Elderly. J. Bone Jt. Surg. Am. Vol..

[23] Giangregorio L.M., Papaioannou A., Macintyre N.J., Ashe M.C., Heinonen A., Shipp K., Wark J., McGill S., Keller H., Jain R. (2014). Too Fit To Fracture: Exercise recommendations for individuals with osteoporosis or osteoporotic vertebral fracture. Osteoporos. Int..

[24] Williamson A., Hoggart B. (2005). Pain: A review of three commonly used pain rating scales. J. Clin. Nurs..

[25] Ware J.E. (2000). SF-36 health survey update. Spine (Phila Pa 1976).

[26] Cook D.J., Guyatt G.H., Adachi J.D., Epstein R.S., Juniper E.F., Austin P.A., Clifton J., Rosen C.J., Kessenich C.R., Stock J.L. (1999). Development and validation of the mini-osteoporosis quality of life questionnaire (OQLQ) in osteoporotic women with back pain due to vertebral fractures. Osteoporosis Quality of Life Study Group. Osteoporos. Int..

[27] Shumway-Cook A., Brauer S., Woollacott M. (2000). Predicting the probability for falls in community-dwelling older adults using the Timed Up & Go Test. Phys. Ther..

[28] Nola G., Mostardini C., Salvi C., Ercolani A.P., Ralli G. (2010). Validity of Italian adaptation of the Dizziness Handicap Inventory (DHI) and evaluation of the quality of life in patients with acute dizziness. Acta Otorhinolaryngol. Ital..

[29] Martines F., Salvago P., Dispenza F., Rizzo S., Letizia Mauro G., Puglisi S. (2019). Treatment with a new nutraceutical compound on patients suffering from balance disorders: Dizziness handicap inventory scores. Acta Med. Mediterr..

[30] Silverman S., Viswanathan H.N., Yang Y.C., Wang A., Boonen S., Ragi-Eis S., Fardellone P., Gilchrist N., Lips P., Nevitt M. (2011). Impact of clinical fractures on health-related quality of life is dependent on time of assessment since fracture: Results from the FREEDOM trial. Osteoporos. Int..

[31] Adachi J.D., Ioannidis G., Olszynski W.P., Brown J.P., Hanley D.A., Sebaldt R.J., Petrie A., Tenenhouse A., Stephenson G.F., Papaioannou A. (2002). The impact of incident vertebral and non-vertebral fractures on health related quality of life in postmenopausal women. BMC Musculoskelet. Disord..

[32] Stanghelle B., Bentzen H., Giangregorio L., Pripp A.H., Bergland A. (2019). Associations between health-related quality of life, physical function and pain in older women with osteoporosis and vertebral fracture. BMC Geriatr..

[33] Makarova E.V., Marchenkova L.A., Eryomushkin M.A., Styazkina E.M., Chesnikova E.I. (2020). Balance and muscle strength tests in patients with osteoporotic vertebral fractures to develop tailored rehabilitation programs. Eur. J. Transl. Myol..

[34] Iolascon G., Giamattei M.T., Moretti A., Di Pietro G., Gimigliano F., Gimigliano R. (2013). Sarcopenia in women with vertebral fragility fractures. Aging Clin. Exp. Res..

[35] Tetsunaga T., Tetsunaga T., Nishida K., Tanaka M., Sugimoto Y., Takigawa T., Takei Y., Ozaki T. (2017). Denosumab and alendronate treatment in patients with back pain due to fresh osteoporotic vertebral fractures. J. Orthop. Sci..

[36] Rolvien T., Schmidt T., Butscheidt S., Amling M., Barvencik F. (2017). Denosumab is effective in the treatment of bone marrow oedema syndrome. Injury.

[37] Dufresne S.S., Dumont N.A., Bouchard P., Lavergne E., Penninger J.M., Frenette J. (2015). Osteoprotegerin protects against muscular dystrophy. Am. J. Pathol..

[38] Dufresne S.S., Dumont N.A., Boulanger-Piette A., Fajardo V.A., Gamu D., Kake-Guena S.A., David R.O., Bouchard P., Lavergne É., Penninger J.M. (2016). Muscle RANK is a key regulator of Ca^2+^ storage, SERCA activity, and function of fast-twitch skeletal muscles. Am. J. Physiol. Cell Physiol..

[39] Kramer I.F., Snijders T., Smeets J.S.J., Leenders M., van Kranenburg J., den Hoed M., Verdijk L.B., Poeze M., van Loon L.J.C. (2017). Extensive Type II Muscle Fiber Atrophy in Elderly Female Hip Fracture Patients. J. Gerontol. A Biol. Sci. Med. Sci..

[40] Chotiyarnwong P., McCloskey E., Eastell R., McClung M.R., Gielen E., Gostage J., McDermott M., Chines A., Huang S., Cummings S.R. (2020). A Pooled Analysis of Fall Incidence From Placebo-Controlled Trials of Denosumab. J. Bone Miner. Res..

[41] Gibbs J.C., MacIntyre N.J., Ponzano M., Templeton J.A., Thabane L., Papaioannou A., Giangregorio L.M. (2019). Exercise for improving outcomes after osteoporotic vertebral fracture. Cochrane Database Syst. Rev..

[42] Ziebart C., Gibbs J.C., McArthur C., Papaioannou A., Mittmann N., Laprade J., Kim S., Khan A., Kendler D.L., Wark J.D. (2019). Are osteoporotic vertebral fractures or forward head posture associated with performance-based measures of balance and mobility?. Arch Osteoporos..

[43] Rizzo S., Sanfilippo V., Terrana P., Scaturro D., Lauricella L., Letizia Mauro G. (2019). Presbyastasis: From diagnosis to management. Sensorineural Hearing Loss: Pathophysiology, Diagnosis and Treatment.

[44] Watson S.L., Weeks B.K., Weis L.J., Harding A.T., Horan S.A., Beck B.R. (2019). High-intensity exercise did not cause vertebral fractures and improves thoracic kyphosis in postmenopausal women with low to very low bone mass: The LIFTMOR trial. Osteoporos. Int..

[45] Berk E., Koca T.T., Güzelsoy S.S., Nacitarhan V., Demirel A. (2019). Evaluation of the relationship between osteoporosis, balance, fall risk, and audiological parameters. Clin. Rheumatol..

[46] Kerr C., Bottomley C., Shingler S., Giangregorio L., de Freitas H.M., Patel C., Randall S., Gold D.T. (2017). The importance of physical function to people with osteoporosis. Osteoporos. Int..

